# Utility of CD138/syndecan-1 immunohistochemistry for localization of plasmacytes is tissue-dependent in B6 mice

**DOI:** 10.1186/s13104-022-06100-5

**Published:** 2022-06-25

**Authors:** David K. Meyerholz, Mariah R. Leidinger, J. Adam Goeken, Thomas R. Businga, Allison Akers, Sebastian Vizuett, Courtney A. Kaemmer, Jordan L. Kohlmeyer, Rebecca D. Dodd, Dawn E. Quelle

**Affiliations:** 1 Department of Pathology, University of Iowa, Iowa City, IA USA; 2 Department of Neuroscience and Pharmacology, University of Iowa, Iowa City, IA USA; 3 Department of Internal Medicine, University of Iowa, Iowa City, IA USA

**Keywords:** B-cells, CD138/syndecan-1, Immunohistochemistry, Kappa light chain, Plasmacytes

## Abstract

**Objective:**

Inflammation is present in many diseases and identification of immune cell infiltration is a common assessment. CD138 (syndecan-1) is a recommended immunohistochemical marker for human plasmacytes although it is also expressed in various epithelia and tumors. Similarly, CD138 is a marker for murine plasmacytes, but its tissue immunostaining is not well-defined. Endogenous CD138 expression is an important confounding factor when evaluating plasmacyte infiltration. We studied two plasmacyte markers (CD138 and Kappa light chains) for endogenous immunostaining in five organs and one tumor from B6 mice.

**Results:**

Plasmacytes in Peyer’s patches were positive for CD138 and Kappa markers without endogenous immunostaining. Endogenous CD138 immunostaining was widespread in liver, kidney, lung and a malignant peripheral nerve sheath tumor (MPNST) versus regionalized immunostaining in skin and small intestine wall. Endogenous Kappa immunostaining was absent in all tissues except for plasmacytes. Tissues with widespread endogenous CD138 immunostaining were contrasted by absence of endogenous Kappa immunostaining. Here, plasmacytes would not be distinguished by CD138, but would be obvious by Kappa immunostaining. Our study suggests that utility of immunostaining for plasmacytes by CD138 is tissue dependent in mice. Additionally, Kappa immunostaining may be a useful alternative in mouse tissues with confounding endogenous CD138 immunostaining.

## Introduction

Inflammation is a pathologic feature in many conditions including but not limited to infectious [1], genetic [2], metabolic [3] and cancer [4, 5] etiologies. Therefore, understanding the contributions of inflammation to disease pathogenesis is a meaningful component of biomedical studies. Several approaches can be used to study inflammation in tissues, each with advantages and limitations [6]. Immunohistochemistry is a common technique that is useful to identify expression of specific markers to localize immune cell infiltration in tissues [7–10]. Immunostaining can be used to determine immune cell location, distribution, activation state, extent of infiltration (qualitative, semi-quantitative, quantitative), and corroborate infiltration with clinical data [6, 7, 11–13].

Plasmacytes (also known as plasma cells) are fully differentiated B cells able to synthesize and secrete immunoglobulins [14]. Plasmacytes are defined by the expression of several protein markers in mice and humans [15], but the value of these individual markers for study can be technique dependent. For instance, CD138 (syndecan-1) is recommended as an immunohistochemical tissue marker specific for plasmacytes in humans and mice [10, 15–17]. CD138 is member of the syndecan family that are characterized by three structural components: extracellular, transmembrane and intracellular domains [18]. The transmembrane configuration of CD138 reflects its importance in cell-to-cell and matrix-to-cell interactions. In human tissues, CD138 immunostaining is seen in plasmacytes and several epithelial tissues, and is increasingly recognized in several types of human cancer [18, 19]. In mouse tissues, CD138 immunostaining is seen in plasmacytes, but published images of these are often constrained to tissues devoid of epithelium (e.g., lymphoid tissues) [16, 17]. In contrast to humans, endogenous CD138 expression in healthy mouse organs or even in cancers (except plasmacyte cancers) are not defined by immunostaining studies [10, 16, 17].

The diagnostic ability to detect specific cellular staining (histochemical or immunohistochemistry) is limited by the extent and intensity of endogenous immunostaining in the experimental tissues [20–22]. Ideally, target immune cells expressing the marker should have strong specific immunostaining while the adjacent off-target cells should lack immunostaining. These staining differences provide the contrast needed for successful tissue studies using morphology, scoring or digital analysis [23].

In this study, we conducted two parallel investigations. First, CD138 expression was evaluated by immunostaining to define endogenous cellular/tissue localization in select healthy mouse organs. We also had access to a malignant peripheral nerve sheath tumor (MPNST) and this cancer tissue was also evaluated. In the second investigation, serial tissue sections were immunostained for a different plasmacyte marker, Kappa light chains (Kappa). We then compared the extent of endogenous immunostaining for each marker to determine how it might impact detection of plasmacytes.

## Main text

### Methods

Mouse tissues were acquired from archival tissue repositories (Comparative Pathology Laboratory, University of Iowa) that originated from studies approved by University of Iowa Animal Care and Use Committee following published guidelines for animal care and use. Use of archival tissue repositories avoided the need for live mice to be studied. Organs (lungs, intestine, liver, kidney and skin) from male and female (3/sex) mice were studied. Inclusion criteria for mice included: adult (sexually mature over  ~ 8 weeks of age) and record of wildtype “B6” strain. Additionally, tissue blocks from a mouse with a malignant peripheral nerve sheath tumor was identified. The mouse had been treated with anti-tumor drugs [Palbociclib (100 mg/kg) and Mirdametinib (1 mg/kg), daily oral gavage] for 15 days prior to harvest. The tumor was induced by CRISPR/Cas9 editing of *Nf1*, *Ink4a* and *Arf* genes in the sciatic nerve of a wild-type C57BL/6 mouse, as describe [24–26].

All tissues were formalin-fixed and paraffin-embedded. Tissues sectioned (~ 4 µm) onto glass slides and hydrated through a series of progressive xylenes and ethanol baths. Immunohistochemistry of markers was optimized for detection of plasmacytes in lymph node. CD138/syndecan-1 was performed as previously described [27]. Briefly, heat-induced antigen retrieval (Tris buffer pH 9.0, 125 °C × 5 min) was performed followed by a series of tissue blocks [3% hydrogen peroxide × 8 min, avidin/biotin blocking kit (#SP-2001, Vector Laboratories), and Rodent Block M kit (Biocare Medical)]. Primary antibody (1:3000 × 1 h, rat anti-mouse monoclonal, clone 281-2, Cat#553712, BD Pharmingen Company) was applied followed by secondary antibody [biotinylated rabbit anti-rat IgG, (Vector Laboratories BA-4001)] and ABC kit (PK-6100, Vectastain Elite ABC kit). For Kappa light chains (Kappa), antigen retrieval (Tris buffer pH 9.0, 125 °C × 5 min) was performed followed by a series of tissue blocks (3% hydrogen peroxide × 8 min and 10% goat serum × 30 min). Primary antibody against Kappa (1:400 × 1 h, rabbit monoclonal, clone RM103, #SAB5600201, Sigma Aldrich) was applied followed by Envision-Plus HRP Rabbit kit (Agilient). For both markers, diaminobenzidine (DAB, brown color) was applied as the chromogen, tissues were counter-stained with Harris hematoxylin (blue color) and cover slipped.

For each type of mouse tissue, immunostaining for the two markers were examined by a boarded veterinary pathologist in a post-examination masked manner [28]. Tissues were qualitatively evaluated [23] using tissue morphology to identify immunostain localization by each marker. Immunostaining intensity was defined as negative (lacking obvious stain);  + (weak brown stain); or  ++  (moderate to strong stain i.e. partially to fully obscuring detection of the counterstain). These results were summarily reported as representative of the groups, but if any differences were noted, these were reported in more detail. Representative images were collected (BX53 microscope, DP73 digital camera and CellSens Dimension Software, Olympus).

### Results

**Fig. 1:**
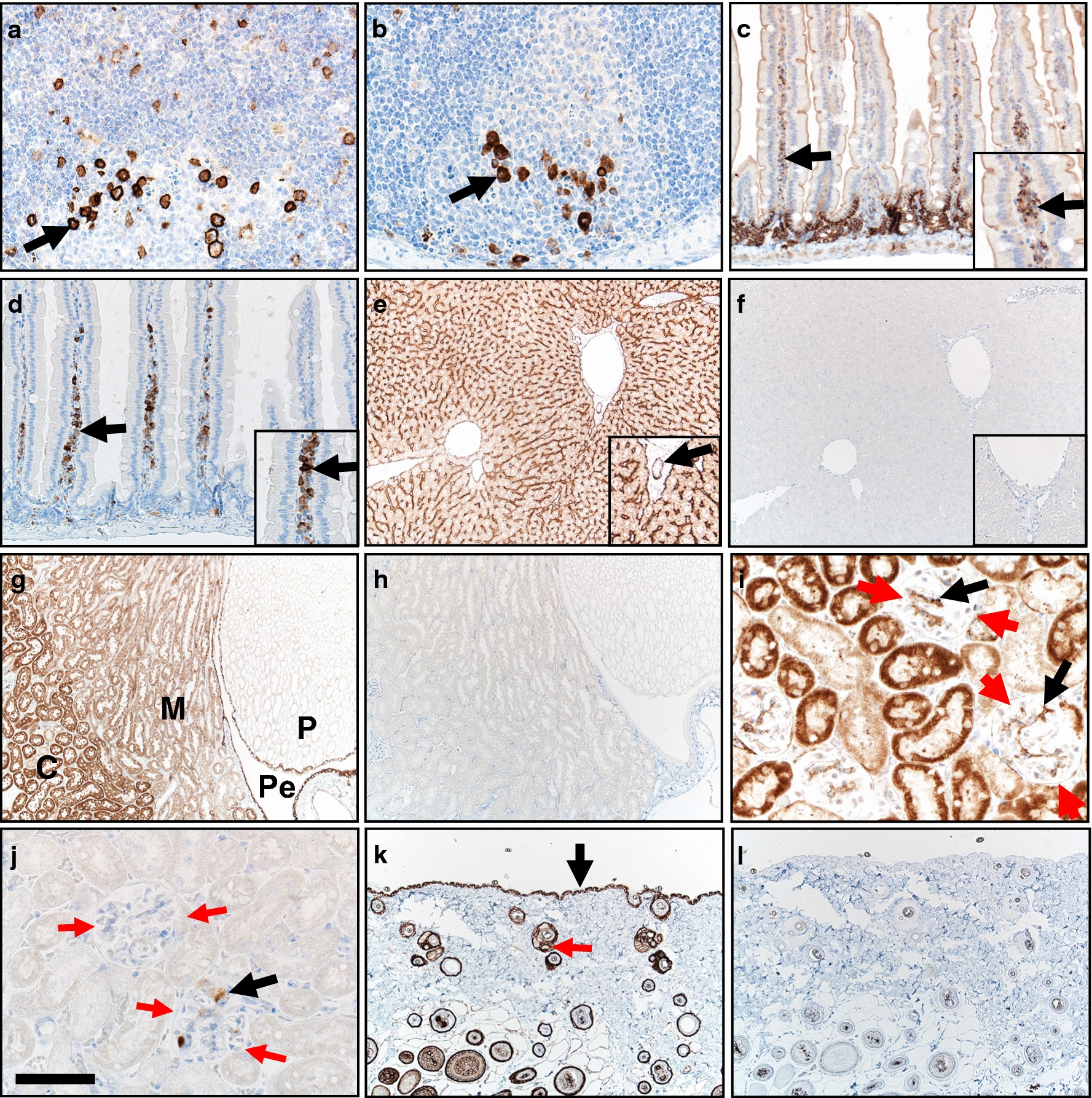
Immunostaining for CD138 (**a**, **c**, **e**, **g**, **i**, **k**) and Kappa (**b**, **d**, **f**, **h**, **j**, **l**) markers in B6 mouse tissues. **a**, **b** Small intestinal Peyer’s patches had plasmacyte immunostaining (arrows) for both markers. **c**, **d** Small intestine wall. Plasmacytes (arrows and inset) were immunostained in lamina propria of villi for both markers. **e** Liver. CD138 immunostaining was localized in a sinusoidal pattern along with immunostaining of hepatocytes and biliary duct epithelium (arrow, inset). **f** Liver. Kappa immunostaining was negative (inset). **g** Kidney. Widespread CD138 immunostaining was seen in cortex(C), medulla (M), papilla (P) and pelvis (Pe) regions. **h** Kappa immunostaining was negative. **i**, **j** Renal glomeruli (highlighted by red arrows) had immunostaining of cells (black arrows). **k** Skin. CD138 immunostaining of epidermal (black arrow) and follicular epithelium (red arrow). **l** Skin. Kappa was negative immunostaining. DAB chromogen (brown color) and hematoxylin counter stain (blue color), bar = 250 µm (**e**–**h**, **k**, **l**), 125 µm (**c**, **d**), and 62 µm (**a**, **b**, **i**, **j**)

In the small intestine, Peyer’s patches are secondary lymphoid organs that appear along the serosal border [29]. The Peyer’s patches were initially examined for CD138 and Kappa immunostaining. Both markers immunostained plasmacyte aggregates within and adjacent to germinal centers (Fig. 1a, b; Table 1), while the lymphoid tissue was negative. Plasmacytes are also commonly localized to the lamina propria of small intestinal villi [30]. Here, plasmacytes were strongly stained by CD138 and Kappa (Fig. 1c, d). Intestinal epithelium showed regional variability of CD138 immunostaining that was consistently stronger in the crypt than in the villus enterocytes (Fig. 1c). Kappa immunostaining of the small intestine epithelium was negative (Fig. 1d).

**Table 1:** CD138 and Kappa immunostaining patterns in various tissues

Organ/tissue	CD138	Kappa
Small intestine		
Plasmacytes	Peyer’s patches (++) Lamina propria of villi (++)	Peyer’s patches (++) Lamina propria of villi (++)
Enterocytes	Enterocytes (crypts > surface) (Neg to ++)	(Neg)
Liver		
Hepatocytes	Sinusoidal pattern/basolateral hepatocytes (++) Hepatocyte cytoplasm (+)	(Neg)
Bile duct	Biliary epithelium (++)	(Neg)
Kidney		
Glomeruli	Bowman’s capsule (+) Cellular immunostaining common (++)	(Neg) Cellular immunostaining rare to scattered, detected in only 4 of 6 animals (+/++)
Tubules/ducts	Cortex > medulla (+/++)	(Neg)
Pelvis	Urothelium (++)	(Neg)
Skin		
Epidermis	(++)	(Neg)
Follicles/adnexa	Follicular epithelium (++) Sebaceous glands (++)	(Neg)
Lung		
Airways	Surface epithelium (++)	(Neg)
Alveolar epithelium	Type 2 cells (++) Type 1 cell (+)	(Neg)

(Staining intensity) = *Neg* negative; *+* weak; *++* moderate to strong

In the liver, CD138 immunostaining was prominent in a sinusoidal pattern that has been reported in humans as localization to the basolateral surface of hepatocytes [31] (Fig. 1e; Table 1). Additionally, CD138 immunostaining was mild in cytoplasm of hepatocytes and moderate in bile duct epithelium. Kappa immunostaining was negative (Fig. 1f).

In the kidney (Fig. 1g–j; Table 1), CD138 immunostaining was widespread in tubules/ducts with stronger staining in the cortex compared to the medulla and papilla. In the renal pelvis, CD138 immunostaining was localized to the lining urothelium (Fig. 1g). Glomeruli had CD138 immunostaining localized to Bowman’s capsule with scattered cells staining in all glomeruli (Fig. 1i). Kappa immunostaining (Fig. 1h, j) was completely negative in two of six animals (Fig. 1h), but in four of the six animals Kappa immunostaining was localized to a few cells in multifocal glomeruli (Fig. 1j). Plasmacytes have been observed in glomeruli as part of chronic kidney diseases in humans [32]. Discrete plasmacyte aggregates were seen by Kappa immunostaining but were obscured by endogenous CD138 immunostaining.

In the skin, CD138 immunostaining was seen in epidermis and follicular epithelium/adnexa (Fig. 1k; Table 1). Kappa immunostaining was consistently negative (Fig. 1l).

**Fig. 2:**
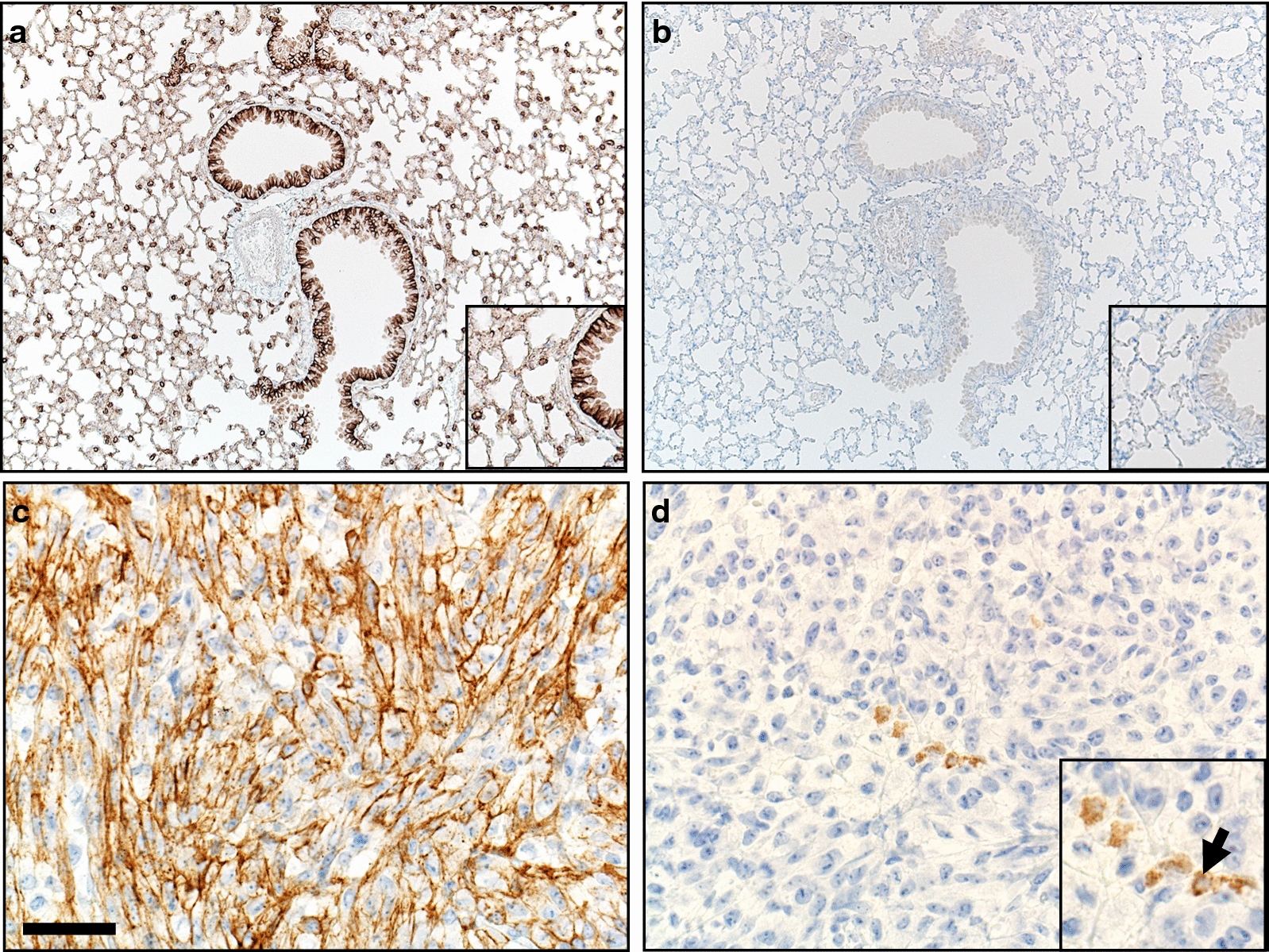
Immunostaining for CD138 and Kappa markers in B6 mouse tissues. **a** Lung had diffuse CD138 immunostaining of airway surface epithelium and alveolar type 2 and 1 epithelial cells (inset). **b** Lung. Kappa immunostaining was negative (inset). **c**, **d** Malignant peripheral nerve sheath tumor. Neoplastic cells had widespread CD138 immunostaining (**c**), whereas Kappa was negative in the tumor except for discrete small cellular aggregates of plasmacytes (**d**, inset, arrow). DAB chromogen (brown color) and hematoxylin counter stain (blue color), bar = 160 µm (a,b) and 40 µm (**c**, **d**)

In the lung, CD138 immunostaining was localized to airway surface epithelium as well as alveolar epithelia (type 2 cells > type 1 cells) (Fig. 2a; Table 1). Kappa immunostaining was consistently negative (Fig. 2b).

In a murine MPNST, plasmacyte markers were evaluated (Fig. 2c, d; Table 1). CD138 immunostaining had widespread localization to most cancer cells, obscuring any plasmacytes (Fig. 2c). In contrast, Kappa immunostaining was consistently negative in cancer cells and this allowed for discrete detection of small plasmacyte aggregates within the tumor (Fig. 2d).

### Discussion

We evaluated CD138 immunostaining in healthy mouse tissues to determine normal endogenous expression and whether it could possibly confound evaluation for CD138^+^ plasmacytes. We found that endogenous CD138 immunostaining was tissue dependent in mice. Lymphoid tissues of the Peyer’s patches lacked immunostaining, making the identification and specific localization of CD138^+^ plasmacytes straightforward. The contrast between positive and negative immunostaining tissue explains why CD138 is useful for plasmacyte detection in lymphoid tissues in humans and mice [10, 17, 33, 34]. For CD138, kidney, liver, lung and even a MPNST tumor had widespread immunostaining, while skin and small intestine had more regionalized epithelial staining. Our findings suggest that widespread endogenous CD138 immunostaining in experimental tissues and tumors could limit the diagnostic analysis for CD138^+^ plasmacytes [20]. Wide expression of CD138 in nonhematopoietic murine tissues was reported in one study, but immunostaining had not been evaluated [35]. Surprisingly, many studies for localization of CD138^+^ plasmacytes in mouse tissues don’t mention observed endogenous CD138 expression in epithelial cells [36]. Conversely, mouse studies investigating epithelial expression of CD138 often fail to mention potential for infiltrative plasmacyte immunostaining to affect tissue analysis [37–41]. Several of these same epithelial-focused studies exclusively use “syndecan-1” but avoid use of “CD138” terminology; the reverse bias in terminology use is also common in plasmacyte studies. Inconsistent use of marker terminology can confuse researchers reading the published literature. These results suggest more consistency in marker terminology along with transparent discussion of expected to observed tissue distribution by markers should be included in immunohistochemical studies.

A second aim of this study was to evaluate Kappa as an alternative plasmacyte marker in serial sections of the same mouse tissues. Kappa light chains are components of antibodies produced by B cells/plasmacytes and may play a role in immunity and self-tolerance [42, 43]. We found that endogenous immunohistochemical expression in mouse tissues was broadly negative except for observation of Kappa^+^ cells morphologically consistent with plasmacytes in tissues. Interestingly, kidney and MPNST tissues provided clear examples of how widespread endogenous expression could negatively affect detection of plasmacytes. In a different example, the small intestine had discreet plasmacyte immunostaining for both markers in the lamina propria of villi, as expected in the normal intestine. Since endogenous immunostaining of the villus epithelium did not overtly affect identification of the plasmacytes—CD138 could feasibly be used in some types of studies on tissues (small intestine, skin) with regional endogenous expression.

Our study identifies several considerations for researchers studying plasmacytes (or other immune cells) by immunohistochemistry in tissues. (1) Investigational tissues should be evaluated for endogenous expression of candidate markers. If the endogenous expression is sufficiently low or localized—then use of that marker for plasmacyte localization may be feasible. (2) If the endogenous tissue expression of the candidate marker is sufficient to affect plasmacyte detection or analysis then alternative approaches such as a different marker or dual labeling might be needed. (3) If endogenous expression is detected in study tissues, it should be acknowledged for transparency and reproducibility in other studies. (4) Awareness of endogenous tissue expression could have important implications for other techniques too, such as immunofluorescence and whole tissue expression studies. These techniques often rely on assumptions of marker specificity and they lack the ability for secondary quality checks using morphologic corroboration that is available in immunohistochemistry studies from fixed sections. (5) Lastly, this study can help mitigate gaps in the literature about CD138 immunostaining and guide scientists to make more efficient choices regarding their study designs and use of resources (fiscal, labor and reagents).

### Limitations

This study is not without potential limitations. First, we studied select tissues from mice on a B6 background to provide useful examples of staining patterns, but we cannot assume that our results will necessarily be applicable to other tissues or other murine strains or substrains. Second, we focused primarily on healthy tissues, so we cannot rule out that diseased tissues with inflammation or remodeling changes might display differences in cellular localization or intensity using the markers we studied. Lastly, we studied one cancer type—a MPNST that showed widespread immunostaining of tumor cells for CD138. While we cannot make definitive statements about CD138 immunostaining in other mouse cancers, its presence in several human cancers (carcinomas and sarcomas) [18, 19] could suggest CD138 expression in cancers could confound mouse plasmacyte detection and localization.

## Data Availability

The datasets used and/or analyzed during the current study are available from the corresponding author on reasonable request.

## Abbreviations

ABC: Avidin biotin complex
DAB: Diaminobenzidine
HRP: Horseradish peroxidase
MPNST: Malignant peripheral nerve sheath tumor

## References

[1] Zheng J, Meyerholz D, Wong LR, Gelb M, Murakami M, Perlman S (2021). Coronavirus-specific antibody production in middle-aged mice requires phospholipase A2G2D. J Clin Invest.

[2] Ortiz-Munoz G, Yu MA, Lefrancais E, Mallavia B, Valet C, Tian JJ, Ranucci S, Wang KM, Liu Z, Kwaan N (2020). Cystic fibrosis transmembrane conductance regulator dysfunction in platelets drives lung hyperinflammation. J Clin Invest.

[3] Jiang Q, Maresch CC, Petry SF, Paradowska-Dogan A, Bhushan S, Chang Y, Wrenzycki C, Schuppe HC, Houska P, Hartmann MF (2020). Elevated CCL2 causes Leydig cell malfunction in metabolic syndrome. JCI Insight.

[4] Greten FR, Grivennikov SI (2019). Inflammation and cancer: triggers, mechanisms, and consequences. Immunity.

[5] Wang L, Yang H, Dorn P, Berezowska S, Blank F, Wotzkow C, Marti TM, Peng RW, Harrer N, Sommergruber W (2021). Peritumoral CD90+CD73+ cells possess immunosuppressive features in human non-small cell lung cancer. EBioMedicine.

[6] Meyerholz DK, Sieren JC, Beck AP, Flaherty HA (2018). Approaches to evaluate lung inflammation in translational research. Vet Pathol.

[7] Donovan KM, Leidinger MR, McQuillen LP, Goeken JA, Hogan CM, Harwani SC, Flaherty HA, Meyerholz DK (2018). Allograft inflammatory factor 1 as an immunohistochemical marker for macrophages in multiple tissues and laboratory animal species. Comp Med.

[8] Meyerholz DK, Ofori-Amanfo GK, Leidinger MR, Goeken JA, Khanna R, Sieren JC, Darbro BW, Quelle DE, Weimer JM (2017). Immunohistochemical markers for prospective studies in neurofibromatosis-1 porcine models. J Histochem Cytochem.

[9] Meyerholz DK, Lambertz AM, Reznikov LR, Ofori-Amanfo GK, Karp PH, McCray PB, Welsh MJ, Stoltz DA (2016). Immunohistochemical detection of markers for translational studies of lung disease in pigs and humans. Toxicol Pathol.

[10] Rehg JE, Rahija R, Bush D, Bradley A, Ward JM (2015). Immunophenotype of spontaneous hematolymphoid tumors occurring in young and aging female CD-1 mice. Toxicol Pathol.

[11] Kempuraj D, Twait EC, Williard DE, Yuan Z, Meyerholz DK, Samuel I (2013). The novel cytokine interleukin-33 activates acinar cell proinflammatory pathways and induces acute pancreatic inflammation in mice. PLoS ONE.

[12] Bartlett JA, Ramachandran S, Wohlford-Lenane CL, Barker CK, Pezzulo AA, Zabner J, Welsh MJ, Meyerholz DK, Stoltz DA, McCray PB (2016). Newborn cystic fibrosis pigs have a blunted early response to an inflammatory stimulus. Am J Respir Crit Care Med.

[13] Saha D, Rabkin SD (2020). Immunohistochemistry for tumor-infiltrating immune cells after oncolytic virotherapy. Methods Mol Biol.

[14] Allen HC, Sharma P (2021). Histology, plasma cells.

[15] Brynjolfsson SF, Persson Berg L, Olsen Ekerhult T, Rimkute I, Wick MJ, Martensson IL, Grimsholm O (2018). Long-lived plasma cells in mice and men. Front Immunol.

[16] Rehg JE, Bush D, Ward JM (2012). The utility of immunohistochemistry for the identification of hematopoietic and lymphoid cells in normal tissues and interpretation of proliferative and inflammatory lesions of mice and rats. Toxicol Pathol.

[17] Ward JM, Erexson CR, Faucette LJ, Foley JF, Dijkstra C, Cattoretti G (2006). Immunohistochemical markers for the rodent immune system. Toxicol Pathol.

[18] Palaiologou M, Delladetsima I, Tiniakos D (2014). CD138 (syndecan-1) expression in health and disease. Histol Histopathol.

[19] Kind S, Merenkow C, Buscheck F, Moller K, Dum D, Chirico V, Luebke AM, Hoflmayer D, Hinsch A, Jacobsen F (2019). Prevalence of Syndecan-1 (CD138) expression in different kinds of human tumors and normal tissues. Dis Markers.

[20] Goto K (2016). CD138 expression is observed in the urothelial epithelium and in various urothelial carcinomas, and cannot be evidence for plasmacytoid urothelial carcinoma. Int J Surg Pathol.

[21] Magaki S, Hojat SA, Wei B, So A, Yong WH (2019). An introduction to the performance of immunohistochemistry. Methods Mol Biol.

[22] Meyerholz DK, Beck AP, Goeken JA, Leidinger MR, Ofori-Amanfo GK, Brown HC, Businga TR, Stoltz DA, Reznikov LR, Flaherty HA (2018). Glycogen depletion can increase the specificity of mucin detection in airway tissues. BMC Res Notes.

[23] Meyerholz DK, Beck AP (2018). Principles and approaches for reproducible scoring of tissue stains in research. Lab Invest.

[24] Kohlmeyer JL, Kaemmer CA, Lingo JJ, Voigt E, Leidinger MR, McGivney GR, Scherer A, Koppenhafer SL, Gordon DJ, Breheny P (2022). Oncogenic RABL6A promotes NF1-associated MPNST progression in vivo. Neurooncol Adv.

[25] Huang J, Chen M, Whitley MJ, Kuo HC, Xu ES, Walens A, Mowery YM, Van Mater D, Eward WC, Cardona DM (2017). Generation and comparison of CRISPR-Cas9 and Cre-mediated genetically engineered mouse models of sarcoma. Nat Commun.

[26] Scherer A, Stephens VR, McGivney GR, Gutierrez WR, Laverty EA, Knepper-Adrian V, Dodd RD (2020). Distinct tumor microenvironments are a defining feature of strain-specific CRISPR/Cas9-induced MPNSTs. Genes (Basel).

[27] Tompkins VS, Sompallae R, Rosean TR, Walsh S, Acevedo M, Kovalchuk AL, Han SS, Jing X, Holman C, Rehg JE (2016). Transgenic mouse model of IgM(+) lymphoproliferative disease mimicking Waldenstrom macroglobulinemia. Blood Cancer J.

[28] Meyerholz DK, Beck AP (2018). Fundamental concepts for semiquantitative tissue scoring in translational research. ILAR J.

[29] De Jesus M, Ahlawat S, Mantis NJ (2013). Isolating and immunostaining lymphocytes and dendritic cells from murine Peyer’s patches. J Vis Exp.

[30] Spencer J, Sollid LM (2016). The human intestinal B-cell response. Mucosal Immunol.

[31] Regos E, Karaszi K, Reszegi A, Kiss A, Schaff Z, Baghy K, Kovalszky I (2020). Syndecan-1 in liver diseases. Pathol Oncol Res.

[32] Houghton DC, Troxell ML (2011). An abundance of IgG4+ plasma cells is not specific for IgG4-related tubulointerstitial nephritis. Mod Pathol.

[33] Steiniger BS, Raimer L, Ecke A, Stuck BA, Cetin Y (2020). Plasma cells, plasmablasts, and AID(+)/CD30(+) B lymphoblasts inside and outside germinal centres: details of the basal light zone and the outer zone in human palatine tonsils. Histochem Cell Biol.

[34] O'Connell FP, Pinkus JL, Pinkus GS (2004). CD138 (syndecan-1), a plasma cell marker immunohistochemical profile in hematopoietic and nonhematopoietic neoplasms. Am J Clin Pathol.

[35] von Allmen CE, Bauer M, Dietmeier K, Buser RB, Gwerder M, Muntwiler S, Utzinger S, Saudan P, Bachmann MF, Beerli RR (2008). Identification of Ly-6K as a novel marker for mouse plasma cells. Mol Immunol.

[36] Simpson-Abelson MR, Sonnenberg GF, Takita H, Yokota SJ, Conway TF, Kelleher RJ, Shultz LD, Barcos M, Bankert RB (2008). Long-term engraftment and expansion of tumor-derived memory T cells following the implantation of non-disrupted pieces of human lung tumor into NOD-scid IL2Rgamma(null) mice. J Immunol.

[37] Lu Z, Song N, Shen B, Xu X, Fang Y, Shi Y, Ning Y, Hu J, Dai Y, Ding X (2018). Syndecan-1 shedding inhibition to protect against ischemic acute kidney injury through HGF target signaling pathway. Transplantation.

[38] Stepp MA, Gibson HE, Gala PH, Iglesia DD, Pajoohesh-Ganji A, Pal-Ghosh S, Brown M, Aquino C, Schwartz AM, Goldberger O (2002). Defects in keratinocyte activation during wound healing in the syndecan-1-deficient mouse. J Cell Sci.

[39] Yablecovitch D, Shabat-Simon M, Aharoni R, Eilam R, Brenner O, Arnon R (2011). Beneficial effect of glatiramer acetate treatment on syndecan-1 expression in dextran sodium sulfate colitis. J Pharmacol Exp Ther.

[40] Chen S, He Y, Hu Z, Lu S, Yin X, Ma X, Lv C, Jin G (2017). Heparanase Mediates Intestinal Inflammation and Injury in a Mouse Model of Sepsis. J Histochem Cytochem.

[41] Zhang D, Han S, Zhou Y, Qi B, Wang X (2020). Therapeutic effects of mangiferin on sepsis-associated acute lung and kidney injuries via the downregulation of vascular permeability and protection of inflammatory and oxidative damages. Eur J Pharm Sci.

[42] Collins AM, Watson CT (2018). Immunoglobulin light chain gene rearrangements, receptor editing and the development of a self-tolerant antibody repertoire. Front Immunol.

[43] Townsend CL, Laffy JM, Wu YB, Silva O'Hare J, Martin V, Kipling D, Fraternali F, Dunn-Walters DK (2016). Significant differences in physicochemical properties of human immunoglobulin kappa and lambda CDR3 regions. Front Immunol.

